# Reproductive Biology of Albacore Tuna (*Thunnus alalunga*) in the Western Indian Ocean

**DOI:** 10.1371/journal.pone.0168605

**Published:** 2016-12-21

**Authors:** Zahirah Dhurmeea, Iker Zudaire, Emmanuel Chassot, Maria Cedras, Natacha Nikolic, Jérôme Bourjea, Wendy West, Chandani Appadoo, Nathalie Bodin

**Affiliations:** 1 Department of Biosciences, Faculty of Science, University of Mauritius, Réduit, Mauritius; 2 IRD—research unit MARine Biodiversity, Exploitation & Conservation (MARBEC), Fishing Port, Victoria, Mahé, Seychelles; 3 IKERBASQUE, Basque Foundation for Science, Bilbao, Spain; 4 Seychelles Fishing Authority (SFA), Fishing Port, Victoria, Mahé, Seychelles; 5 Ifremer DOI La Réunion, Le Port, Reunion Island, France; 6 Ifremer—research unit MARine Biodiversity, Exploitation & Conservation (MARBEC), Sète, France; 7 Department of Agriculture Forestry and Fisheries (DAFF), Cape Town, South Africa; 8 Department of Marine and Ocean Science, Fisheries and Mariculture, Faculty of Ocean Studies, University of Mauritius, Réduit, Mauritius; Universita degli Studi di Bari Aldo Moro, ITALY

## Abstract

The reproductive biology of albacore tuna, *Thunnus alalunga*, in the western Indian Ocean was examined through analysis of the sex ratio, spawning season, length-at-maturity (*L*_50_), spawning frequency and fecundity. From 2013 to 2015, a total of 923 female and 867 male albacore were sampled. A bias in sex ratio was found in favor of females with fork length (*L*_F_) < 100 cm. Using histological analyses and gonadosomatic index, spawning was found to occur between 10°S and 30°S, mainly to the east of Madagascar from October to January. Large females contributed more to reproduction through their longer spawning period compared to small individuals. The *L*_50_ (mean ± standard error) of female albacore was estimated at 85.3 ± 0.7 cm *L*_F_. Albacore spawn on average every 2.2 days within the spawning region and spawning months, from November to January. Batch fecundity ranged between 0.26 and 2.09 million oocytes and the relative batch fecundity (mean ± standard deviation) was estimated at 53.4 ± 23.2 oocytes g^-1^ of somatic-gutted weight. The study provides new information on the reproductive development and classification of albacore in the western Indian Ocean. The reproductive parameters will reduce uncertainty in current stock assessment models which will eventually assist the fishery to be sustainable for future generations.

## Introduction

Albacore tuna, *Thunnus alalunga* (Bonnaterre, 1788), is a temperate species widely distributed in the tropical, sub-tropical and temperate zones from 50°N to 40°S [1]. It is one of the most important species of commercial tuna fisheries worldwide representing 6% of the total catch in 2013 [2]. In the Indian Ocean, this species is caught mostly using drifting longlines followed by purse-seines and other gears [3]. Albacore represents a very important food and economic resource for countries in this region. For example, it is the most harvested species by pelagic longliners from Reunion Island after swordfish (*Xiphias gladius*) [4]. Baitboats operating along the southern and western coasts of South Africa and Namibia catch around 4,000 tons (t) of albacore annually [5,6]. Albacore is also very important to the economy of small developing island states such as Mauritius where fishing licenses are issued to some hundred foreign longliners annually, most of which target albacore [7]. Here landings of albacore, which are mostly caught in the exclusive economic zone (EEZ), have increased considerably since 2008, reaching 3,580 t in 2011. Moreover, albacore contributes to the revenue of the local artisanal fishermen who target albacore associated to anchored fish aggregating devices which are set along the coast of the island [7]. Catches of albacore in the Indian Ocean were above the maximum sustainable yield (MSY) in 2013 (43,000 t) but were then recorded to be within MSY limits in 2014 (40,981 t) [3]. The last assessment of the albacore tuna stock in the Indian Ocean indicated that the stock is not overfished and not subject to overfishing despite the existence of uncertainties in the methods of assessment [8].

Reproduction plays a major role in a fish population productivity and, as such, in its resilience to fishing and environmental changes [9,10]. Maturity, fecundity, sex ratio and fish condition are the fundamental factors that affect fish population productivity and are thus important parameters for estimating their reproductive potential [10,11], that is their ability to generate viable eggs in relation to the energy available and parental life expectancy [12]. It has recently been recognized that the spawning stock biomass (*B*_S_), long used as a biological indicator in stock assessments, is not suitable to estimate a stock's reproductive potential [13] as it fails to account for the plasticity in reproductive traits [14]. As an alternative to *B*_S_, reproductive potential indices have been suggested [10,15] that take into account the basic reproductive parameters to improve the stock assessments.

Albacore migrates over vast distances and develops separate groups at particular stages of its life cycle [1]. A boundary has been described at approximately 30°S in the Indian Ocean with immature fish located south of it, while large albacore occurred in warmer waters in the north [16,17]. It was proposed that the distribution of the three life history stages (immature, spawning only, and mature) of albacore coincided with the three main oceanic currents of the Indian Ocean: the monsoon-driven current in the north of 10°S, the subtropical gyre between 10°S and 30°S and the Circumpolar Current south of 30°S [18]. Spawning of albacore in the western Indian Ocean is thought to occur predominantly east of Madagascar between 15°S and 25°S during the first and fourth quarters of each year when sea surface temperature is above 24°C [19]. Both immature and mature individuals exhibit a north-south seasonal migration although immature tend to remain in southern latitudes, while mature ones remain further north [17].

The reproductive biology of albacore tuna is generally poorly known compared with other principal market tuna species [20]. In the Indian Ocean, contrary to the western Atlantic Ocean [21,22], Pacific Ocean [23,24] and Mediterranean Sea [25–27], biological studies are scarce and little new information is available [28]. In the northeast Atlantic, sexual maturity for males and females is reached between 90 and 94 cm fork length (*L*_F_) or at age 5, with no difference noted between the sexes [29]. In the South Pacific, the predicted age at which 50% and 100% of the female population reach sexual maturity was found to be 4.5 years (87 cm *L*_F_) [30] and 7 years (94 cm *L*_F_), respectively [31].

Similar to other tunas, female albacore have asynchronous oocyte development, with oocytes of different development stages present in the ovary at the same time [22]. Albacore has indeterminate annual fecundity [32], that is, the potential annual fecundity is not fixed before the onset of the spawning season and unyolked oocytes continue to be matured and spawned over a protracted spawning period in several batches [33,34]. They were also reported to spawn daily, on average 1.3 days, during peak spawning months in the South Pacific [24].

Currently, reproductive parameters used in stock assessment models for Indian Ocean albacore originate largely from other stocks [8,35]. Sensitivity analyses of related stock assessment models have shown that minor modification of these parameter estimates can profoundly influence reference points for which management advice is based on [36]. As a result, given that differences may exist in the population biology of albacore stocks between oceans, using reproductive parameters from other stocks may have a large impact on the stock assessment of Indian Ocean albacore. It is therefore vital that the knowledge on these parameters is enhanced for albacore in the Indian Ocean so that advanced scientific advice can also be improved for the sustainable management of albacore in the region.

With special focus on females due to the higher importance of maternal role in offspring production [37], the present study aims to fully describe the reproductive biology of albacore tuna from the western Indian Ocean through the assessment of the fundamental traits that characterize the reproductive potential of a fish species: (i) sex ratio, (ii) spawning season and location, (iii) length at 50% maturity (*L*_50_), and (iv) spawning frequency and fecundity. Spatial variability in the first two parameters was also investigated.

## Material and Methods

### Field sampling

**Fig 1:**
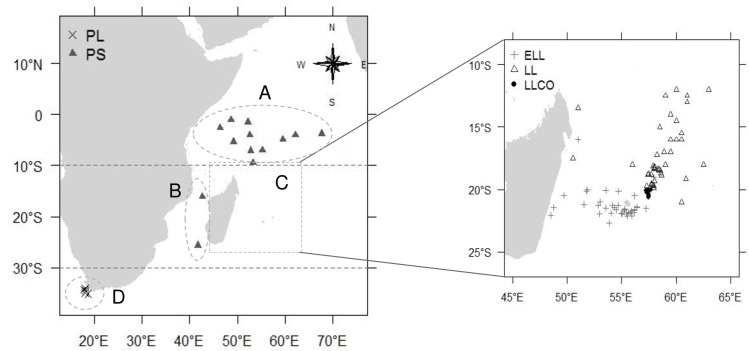
**Map of the western Indian Ocean showing positions of sampled albacore tunas by latitudinal area and region, and by gear.** Seychelles waters located north of 10°S (region A), Mozambique Channel (region B) and Mauritius and Reunion Island waters (region C) both regions located at latitudes 10–30°S, and South Africa waters located south of 30°S (region D). Black crosses: pole-and-liner (PL), grey triangles: purse-seiner (PS), grey crosses: longliner targeting swordfish (ELL), white triangles: deep-freezing longliner (LL), black dots: coastal longliner (LLCO). Regions are separated by grey dotted lines.

A total of 923 female and 867 male albacore, caught from June 2013 to June 2015 between latitudes 10°N-40°S and longitudes 10°E-70°E, were sampled from different regions of the western Indian Ocean (Fig 1; Table 1). Based on the observation of immature, spawning only and mature albacore in the Indian Ocean from previous studies [16,17], the boundaries of latitudes 10°S and 30°S were chosen to assess the monthly variation in the ovarian reproductive phases of albacore tuna in the western Indian Ocean by latitudinal area: latitudes north of 10°S which correspond to the waters of Seychelles (region A), latitudes 10–30°S which group fish caught in the Mozambique Channel (region B) and the waters of Mauritius and Reunion Island (region C), and finally latitudes south of 30°S which include fish from South Africa (region D).

**Table 1:** Number of female and male albacore tuna sampled by region and latitudinal area in the western Indian Ocean.

Latitudinal area	Region	Females		Males	
		*n*	*L*_F_	*n*	*L*_F_
North of 10°S	A	355	94.9 ± 4.4	275	96.8 ± 5.4
10–30°S	B	43	94.1 ± 5.1	49	95.8 ± 5.7
	C	370	98.7 ± 3.8	398	104.0 ± 4.9
South of 30°S	D	155	84.9 ± 6.9	145	85.3 ± 7.8
**Total**		923	94.6 ± 6.7	867	98.1 ± 8 .7

Region A: Seychelles waters, Region B: Mozambique Channel, Region C: Mauritius and Reunion Island waters, Region D: South Africa waters. *n*: number of fish, *L*_F_: mean ± standard deviation in cm.

Albacore from regions A and B were caught by commercial European purse-seiners (PS) landing in Seychelles. Albacore from Reunion Island (region C) were sampled at sea onboard a commercial longliner chartered from November to December 2013, and additional samples were obtained from the catch of longliners targeting swordfish (ELL) from April to August 2014. All vessels from Reunion Island were licensed under the French Ministry of Fisheries and Agriculture. Albacore sampled in Mauritius (region C) were caught from October 2013 to March 2015 by professional artisanal fishermen along the coast of the island using vertical longlines (LLCO) and commercial longliners (LL), and were licensed by the Ministry of Ocean Economy, Marine Resources, Fisheries, Shipping and Outer Islands in Mauritius. Albacore from region D were caught from November 2013 to January 2014 and from April to May 2014 by commercial pole-and-line fishing boats (PL) licensed by the Department of Agriculture, Forestry and Fisheries in South Africa, and part of the fish were sampled at sea by observers. All commercial fishing vessels were authorized to operate in the Indian Ocean Tuna Commission (IOTC) area under Resolution 15/04 and within the EEZ of coastal countries of the western Indian Ocean through fishing agreements. Apart from fish sampled at sea, all the other albacore were sampled at processing plants in compliance with their respective health and safety practices (regions A and B: Victoria in Seychelles, region C: Saint-Denis in Reunion Island, Port-Louis in Mauritius; region D: Cape Town in South Africa) or at fish landing sites (fish caught by LLCO). Since all fish sampled under the present study were already dead by the time of sampling, no ethical approval was required.

Different parameters were recorded for each fish: fork length (*L*_F,_ cm; projected straight distance measured with a caliper from the tip of the upper jaw (snout) to the fork of the tail), pectoral length (*L*_D_, cm; projected straight distance measured with a caliper between the cranial insertion of the pectoral fin and the fork of the tail), total fish weight (*W*_T_, kg), eviscerated fish weight (ie. *W*_T_ excluding the gut, *W*_E_, kg), whole gonad weight trimmed of fat (*W*_G_, g), and sex. Somatic-gutted weight (*W*_S,_ g) was calculated as eviscerated weight (g)—*W*_G._ An estimated *W*_S_ was used where *W*_T_ or *W*_E_ was unavailable but with a known *L*_F_ or *L*_D_ (i.e., regions A and C), by using length-length and length-weight linear regressions estimated by [38]. A 4–5 cm cross-section from the middle third of either ovary was collected randomly for each female fish when available and preserved in neutral 4% buffered formaldehyde for histological analysis [39].

The gonadosomatic index (*I*_G_) [40] was calculated as:

$$I_{G}=\frac{W_{G}}{W_{S}}\times 100$$

### Histological classification of females

A subsample (~0.5 cm) of the ovary cross-section was cut, dehydrated in a graded-series of ethyl-alcohol solutions (70% to absolute), cleared with xylene and infiltrated with paraffin. Paraffin blocks were then sectioned at 6 μm and stained with Harris' hematoxylin and eosin prior to being inspected under a Leica light microscope. Based on the oocyte description and classification criteria by [41] for yellowfin tuna (*Thunnus albacares*), the most advanced oocyte stage in each ovary was identified and used to classify the ovaries into different reproductive phases [42]: the immature phase which is characterized by the presence of oocytes in the primary growth stage (chromatin nucleolus and perinucleolar oocytes), the developing phase which includes oocytes in the cortical alveoli, vitellogenenic 1 and vitellogenic 2 stages, and the spawning capable phase which includes vitellogenic 3, germinal vesicle migration, germinal vesicle breakdown and the hydration stage oocytes (Table 2). When ovulation occurs, the granulosa and theca layers surrounding the hydrated oocyte remain in the ovary tissue and are known as postovulatory follicles (POFs). The presence and the estimated age of POFs were recorded from the histological sections, based on the criteria for yellowfin tuna [43]: 0 hours old (new POFs), <12 hours and >12 hours old.

**Table 2:** Microscopic classification of the ovary of albacore tuna in the western Indian Ocean.

Reproductivephase	MAGO	POFs	Description of atresia in yolked oocytes	Maturity markers	Atretic stage
Immature	PG	Absent	Absent	Absent	-
Developing	CA	Absent	Absent	Absent	-
	Vtg1	Absent	Absent or some atresia may be present	Absent	-
	Vtg2	Absent	Absent or some atresia may be present	Absent	-
Spawning capable	Vtg3	May be present	<50% α atresia. β atresia may be present	May be present	1
	GVM	May be present	<50% α atresia. β atresia may be present	May be present	1
	GVBD	May be present	<50% α atresia. β atresia may be present	May be present	1
	Hyd	Absent	<50% α atresia. β atresia may be present	May be present	1
Regressing	Vtg3	Absent	≥50% α atresia. β atresia may be present	May be present	2
	PG, CA	Absent	100% α atresia. β atresia may be present	May be present	3
	PG, CA	Absent	No α atresia. β atresia present	May be present	4
Regenerating	PG, CA	Absent	Absent	Present	-

MAGO: most advanced group of oocytes, PG: primary growth (chromatin nucleolus and perinucleolar), CA: cortical alveoli, Vtg1: vitellogenic 1, Vtg2: vitellogenic 2, Vtg3: vitellogenic 3, GVM: germinal vesicle migration, GVBD: germinal vesicle breakdown, Hyd: hydration, POFs: postovulatory follicles, α atresia: alpha atresia, β atresia: beta atresia.

The relative intensity of atresia, i.e. the percentage of yolked oocytes in alpha atresia relative to the total number of yolked oocytes, was estimated for each female from the histological section [24]. The presence of maturity markers was recorded such as: unovulated or residual hydrated oocytes and late stages of atresia (gamma or delta) which are referred to as brown or yellow bodies [24].

Spawning capable females, defined as females capable of spawning at sampling time or the near future, had <50% alpha atresia (atretic stage 1). Females were classified in the regressing phase, at the end of their reproductive cycle, if their ovaries contained ≥50% (atretic stage 2) or 100% (atretic stage 3) alpha atresia of yolked oocytes or if only beta atresia was present (atretic stage 4), while the ovaries of regenerating females contained maturity markers but no atresia.

### Length-at-maturity

In this study, fish were considered mature when their ovaries reached the spawning capable phase containing vitellogenic 3 as the most advanced oocytes in addition to those classified in the spawning capable, regressing and regenerating phases [24,44]. The length at which 50% of the population of female albacore is mature (*L*_50_) was derived from fitting the proportion of mature female fish (*P*) to *L*_F_ using the following logistic equation [45]:

$$P=\frac{\exp\left(\alpha +\beta \times L_{F}\right)}{1+\exp\left(\alpha +\beta \times L_{F}\right)}$$

where the parameters *α* and *β* of the regression model were estimated by assuming a binomial distribution for the logit-transformed data of mature female proportion. Fish were grouped into 3-cm classes of *L*_F_ to minimize random fluctuations linked to individual variability. *L*_50_ was computed as -*αβ*^-1^. The variance of the estimate of *L*_50_ was derived from the delta method using a first-order Taylor approximation [46]. A second *L*_50_ was additionally computed where females having oocytes in the cortical alveoli, vitellogenic 1 or vitellogenic 2 as most advanced stage in their ovaries were also considered mature (second criterion), as also assumed in previous studies [41,42].

### Fecundity and spawning frequency

Batch fecundity (*F*_B_; total number of oocytes released per batch) was estimated using a gravimetric method [47] for ovaries in an advanced stage of maturation, i.e. those having migratory nucleus (germinal vesicle migration and germinal vesicle breakdown) or hydration as most advanced oocytes. Oocytes in the hydration stage were large and translucent. Those in the migratory nucleus were observed as being larger than the other yolked oocytes present and had one or several large oil droplets. A hyaline band could also be observed at their border due to the fusion of yolk as stated by [48]. In each preserved portion of ovary, three subsamples of 0.1 ± 0.01 g were retrieved and saturated with glycerin. The number of migratory nucleus or hydrated oocytes in the weighed subsamples was counted under an Olympus stereomicroscope, and the calculated mean number of oocytes per gram was multiplied by *W*_G_ to give an estimate of *F*_B_. The relative batch fecundity (*F*_RB_) was estimated by dividing *F*_B_ by the *W*_S_ of the fish. Assuming that POFs remain visible in the ovary within 24 hours [24,43], the spawning frequency (average interval between successive spawning events) was estimated as the inverse of the spawning fraction. The spawning fraction was calculated as the total number of females with observable POFs divided by the total number of mature females within our sample [49]. In our study, the spawning frequency was estimated during the spawning period excluding frozen samples from deep-freezing longliners in region C since freezing destroys the follicle wall and hence the POFs would be difficult to detect.

### Data analysis

Sex ratio was calculated as the proportion of females to males in the sample and the differences from the expected 1:1 ratio were analyzed by 5-cm *L*_F_ class (to obtain representative number of individuals in each *L*_F_ class) using binomial tests. A Chi-square test (χ^2^) was used to assess the proportion of spawning capable females in small (≤ *L*_F_ of the largest immature fish) and large (> *L*_F_ of the largest immature fish) size classes during the spawning period. Variations in the *I*_G_ with month for each of the three latitudinal areas were tested using analysis of variance (ANOVA) on the log-transformed values to satisfy the assumption of normality. The relationships between the *F*_B_ and *F*_RB_ with other parameters (*L*_F_, *W*_G_, *W*_S_, *I*_G_) were analyzed through linear regressions. All statistical analyses were performed using R version 3.2.2 [50]. All maps were also built in R using the package 'rgdal' [51] and data from http://www.diva-gis.org/Data.

## Results

### Length-frequency and sex ratio

**Fig 2:**
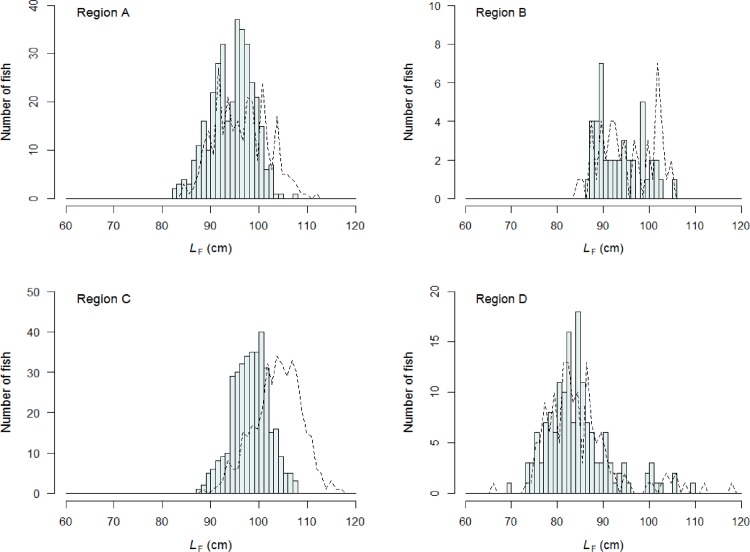
Length-frequency distributions of female and male albacore tuna by region in the western Indian Ocean. Region A: Seychelles waters, Region B: Mozambique Channel, Region C: Mauritius and Reunion Island waters, Region D: South Africa waters. *L*_F_: fork length in cm. Females are represented in light grey bars and males in black dotted lines.

The size of the albacore tuna sampled differed between regions and sexes (Fig 2). Most females from regions A (72%) and B (63%) concentrated in the 90–99 cm *L*_F_ class (range: 83–108 cm *L*_F_) while female albacore caught in region C mainly peaked (82%) in the 95–104 cm *L*_F_ class (range: 88–108 cm *L*_F_). The majority of male albacore from regions A (85%) and B (82%) occupied the 90–104 cm *L*_F_ class and had a maximum *L*_F_ of 112 cm while in region C, most of the males (69%) dominated the 100–109 cm *L*_F_ class and had a maximum *L*_F_ of 117 cm. In region D, both males and females reached smaller sizes compared to the other regions (range: 67–118 cm for males and 70–110 cm for females) with around 60% of the fish concentrating in the 80–89 cm *L*_F_ class.

Analysis of sex ratio showed that female albacore tend to dominate *L*_F_ classes below 100 cm while males dominated in *L*_F_ classes larger than 105 cm (Table 3). In region C, males significantly dominated the larger *L*_F_ classes (105–109 cm and 110–114 cm), while females were dominant in the 90–94 cm and 95–99 cm *L*_F_ classes with the sex ratio significantly different from the expected 1:1 (binomial tests, *P* < 0.05; Table 3). Similar sex ratio results were observed in region A with males significantly dominating 105–109 cm *L*_F_ class while females significantly dominated *L*_F_ classes of 85–89 cm and 95–99 cm. Males and females for regions A and C appeared to occupy a 1:1 ratio in the 100–104 cm *L*_F_ range. In regions B and D, no significant difference in sex ratio was identified in any of the length classes (binomial tests *P* > 0.05; Table 3) although Region B had similar percentage of males across *L*_F_ classes as in Region A.

**Table 3:** Sex ratio of albacore tuna by region in the western Indian Ocean by 5-cm length class.

*L*_F_ class	Region A		Region B		Region C		Region D	
(cm)	*n*	%M	*n*	%M	*N*	%M	*n*	%M
65–69							1	100.0
70–74							5	20.0
75–79							55	49.1
80–84	5	0.0					98	50.0
85–89	64	34.8*^2^	16	43.8	4	25.0	83	45.8
90–94	192	44.0	31	51.6	56	26.8*^1^	29	48.3
95–99	232	35.8*^1^	20	40.0	226	28.3*^1^	7	42.9
100–104	115	57.4	22	72.7	261	48.7	12	41.7
105–109	20	90.0^§^^1^	3	66.7	167	86.8^§^^1^	6	66.7
110–114	2	100.0			39	100.0^§^^1^	2	50.0
115–119					5	100.0	1	100.0

*L*_F_: fork length, %M: percentage of observed males, *n*: total number of fish sampled per *L*_F_ class. Sex ratio analysis was performed through binomial tests. When the *P* value was significant (1: *P* < 0.001, 2: *P*: <0.05), the sex dominating the *L*_F_ class is specified

* = females

^§^ = males.

Region A: Seychelles waters, Region B: Mozambique Channel, Region C: Mauritius and Reunion Island waters, Region D: South Africa waters.

### Size-at-maturity

**Fig 3:**
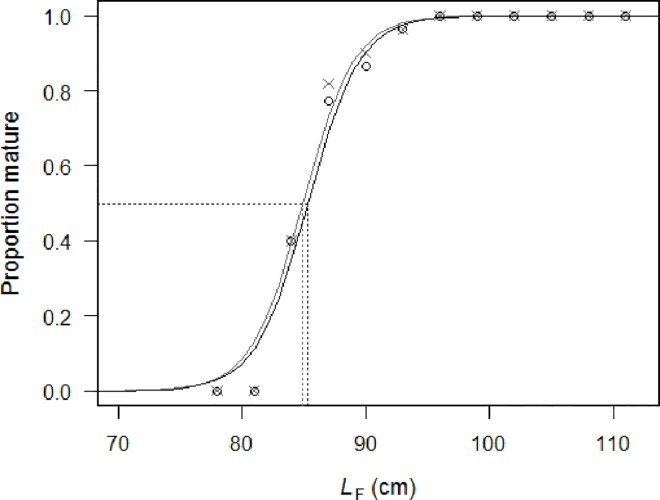
Proportion of mature female albacore in the western Indian Ocean for two different criteria based on 3-cm fork length (*L*_F_) interval fitting. The proportion of females mature when their ovaries contained vitellogenic 3 oocytes onwards (first criterion) are represented by circles and the logistic regression curve fitted to the data is indicated by the black solid line. The proportion of females mature when their ovaries contained cortical alveoli oocytes onwards (second criterion) are represented by grey crosses and the logistic regression curve fitted to the data is indicated by the grey solid line. The horizontal dotted line represents the proportion at which 50% of the population of female albacore tuna is mature and the vertical dotted line shows the corresponding *L*_F_ for each criterion (black and grey for the first and second criteria, respectively).

Most (96%) of the females sampled and analyzed histologically in this study were mature (S1 Table). The largest immature fish (immature phase) was 94 cm *L*_F_ while the smallest mature fish (regenerating phase) was 83 cm *L*_F_. The proportion of mature females fitted the logistic regression model (Fig 3) and allowed for the estimation of *α* (mean ± standard error: -40.8749 ± 6.1068) and *β* (mean ± standard error: 0.4791 ± 0.0686). The *L*_50_ was thus estimated at 85.3 ± 0.7 cm *L*_F_ (*P* < 0.0001, *n* = 665) for female albacore tuna of the western Indian Ocean at the vitellogenic 3 maturity threshold. For the second criterion (when fish containing cortical alveoli oocytes onwards were mature), *α* and *β* were estimated at -40.9305 ± 6.3783 and 0.4820 ± 0.0719 respectively, giving an *L*_50_ of 84.9 ± 0.8 cm *L*_F_ (*P* < 0.0001, *n* = 665).

### Histological classification

Examination of the histological sections revealed that the ovary in the spawning capable phase contains a combination of oocytes in all the different stages of development, confirming an asynchronous ovarian development.

**Fig 4:**
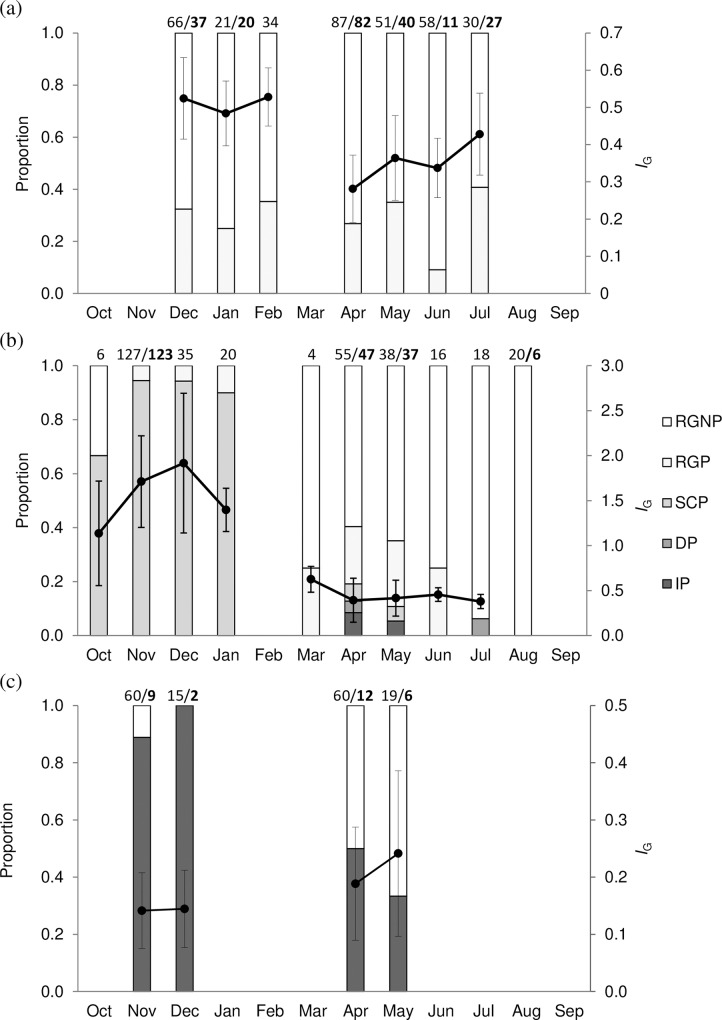
Monthly mean gonadosomatic index and proportion of female albacore with different ovarian developmental phases at different latitudinal areas in the western Indian Ocean. (a) north of 10°S (region A), (b) at latitudes 10–30°S (regions B and C) and (c) south of 30°S (region D) in the western Indian Ocean. *I*_G_: gonadosomatic index, IP: immature phase, DP: developing phase, SCP: spawning capable phase, RGP: regressing phase and RGNP: regenerating phase. Numbers above the bars indicate the numbers of fish analyzed for *I*_G_ and for histological development (in bold) when different. Bars represent standard deviation.

The monthly distribution of fish with ovaries at different reproductive phases was grouped by latitudinal area (north of 10°S, 10–30°S, south of 30°S) in the western Indian Ocean (Fig 4). Most (69%) of the fish caught at latitudes north of 10°S were in the regenerating phase while the rest were in the regressing phase. Around 71% of these regressing females had their ovaries in advanced stages of atresia (atretic stages 3–4). The latitudes 10–30°S were characterized by the occurrence (55%) of spawning capable females (atretic stage 1) which were absent from the higher and lower latitudes. The majority (99%) of these fish were sampled in region C, increasing as from October to November-January (95%), although a small proportion of regressing fish (atretic stage 2) was found during the same period in this region. The abundance of spawning capable fish then decreased from January with very few of them observed in April and May. At the same time, the proportion of both regressing and regenerating females increased. Regressing females in atretic stages 3–4 were observed only from April to July and constituted 76% of all regressing females in this period. The proportion of regenerating females increased progressively from March to dominate completely in August. Finally, south of 30°S, only immature females (with primary growth as most advanced stage) were present except for few regenerating females that occurred mainly during April and May.

Since spawning capable females were found only in region C, the proportion of large (>94 cm *L*_F_, *n* = 223) to small (≤94 cm *L*_F_, *n* = 26) spawning capable females was assessed for this region only. It was found that there was a significantly larger amount of large females in the spawning capable phase (90%) compared to smaller ones (61%) (χ^2^ = 13.7, *P* < 0.001).

Log-transformed *I*_G_ varied significantly between months for fish caught north of 10°S (*F*_(6,340)_ = 36.1, *P* <0.0001), at latitudes 10–30°S (*F*_(8,306)_ = 141.5, *P* <0.0001) and south of 30°S (*F*_(3,150)_ = 3.3, *P* <0.05). Mean *I*_G_ at the latitudes north of 10°S (region A) was found to remain low (*I*_G_ <0.6) throughout all the months sampled with the highest values noted from December (0.52 ± 0.11) to February (0.53 ± 0.08) and the lowest value in April (0.28 ± 0.09) (Fig 4). At latitudes 10–30°S, the highest *I*_G_ were observed from October to January and peaking in November (1.71 ± 0.51) and December (1.92 ± 0.78). These *I*_G_ values coincided to the high percentage of spawning capable females during the same period (October-January) and which also peaked in November and December. *I*_G_ then decreased to <1.0 in March and continued decreasing until July where the lowest mean value was observed (0.38 ± 0.08) corresponding to the presence of regenerating females. Females caught south of 30°S (region D) had the lowest *I*_G_ (*I*_G_ <0.3). The mean *I*_G_ for those females was lowest in November and December (both 0.14 ± 0.07) but increased in April (0.19 ± 0.10) and May (0.24 ± 0.14) owing to the presence of a higher percentage of large mature regenerating female albacore in the region.

### Spawning frequency and fecundity

POFs were observed in ovaries of 55 spawning capable females only from region C and from November to January except in ovaries of three fish caught in April and May. These POFs were observed almost exclusively in ovaries containing vitellogenic 3 as most advanced group of oocytes (96%) and the remaining in two ovaries containing migratory nucleus oocyte stage. In addition, the POFs observed in these ovaries were either <12 hours or >12 hours old. Most of the POFs were observed (84%) in spawning capable fish that were landed fresh by artisanal longliners (LLCO and ELL) while a minority in frozen fish from the industrial longliners (LL). This confirms our reasoning of excluding frozen ovaries for the spawning frequency calculations. Out of the 159 mature fish caught during the spawning period (January-December) for the whole region (excluding longliners in region C), a total of 46 females were found to contain POFs which gave a spawning fraction of 0.29 and a mean interval of 3.5 days.

In region C, where the main spawning activity occurs, a total of 101 mature fish were found during the spawning period among which 46 females contained POFs in their ovaries. Thus, in region C, the spawning fraction was 0.46 and the mean interval was 2.2 days.

**Fig 5:**
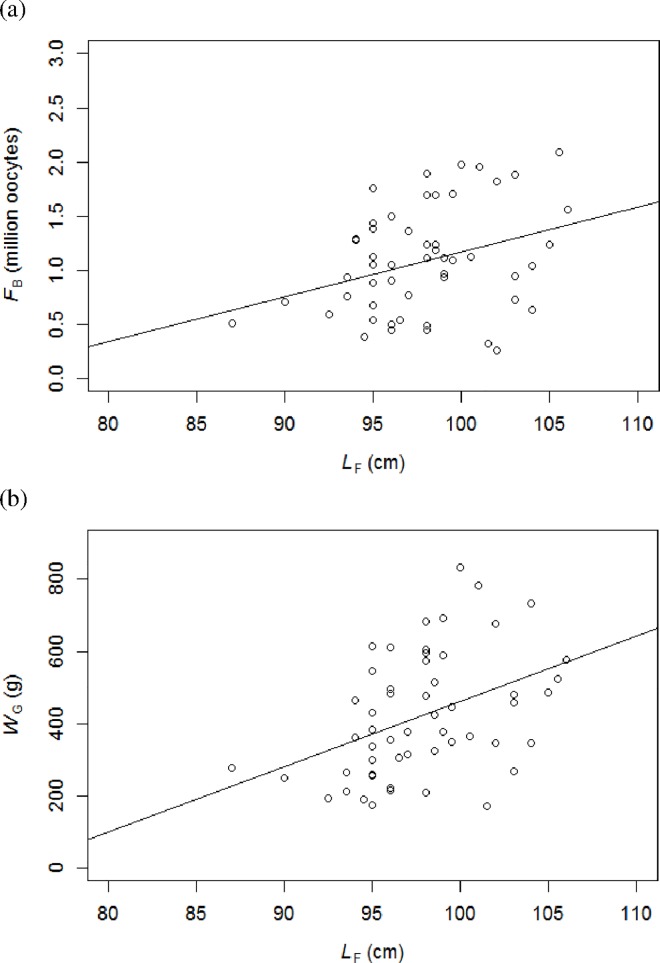
**Relationships between (a) fork length (*L***_**F**_**, cm) and batch fecundity (*F***_**B**_ **in million oocytes) and (b) *L***_**F**_ **and total gonad weight (*W***_**G**_**, g) for albacore tuna in the western Indian Ocean.** *n* = 54.

A total of 54 female fish (range: 87–106 cm *L*_F_) were used for estimating *F*_B_ of albacore tuna in the western Indian Ocean. Among those females, 11 had ovaries at germinal vesicle migration, 19 had germinal vesicle breakdown and 24 had hydration as most advanced oocyte stage. These fish were caught at latitude 10–30°S and only from region C except for one fish from region B. *F*_B_ estimates for the 54 females varied from 0.26 to 2.09 million eggs (mean ± standard deviation: 1.07 ± 0.49 million eggs). *F*_RB_ was estimated to be between 11.1 and 100.5 oocytes g^-1^ of somatic-gutted weight (mean ± standard deviation: 53.4 ± 23.2 oocytes g^-1^). Linear regressions showed no significant relationship between *F*_B_ and *F*_RB_ with *W*_S_ (Table 4). However, a highly significant positive linear relationship was observed between *W*_G_ and *I*_G_ with both *F*_B_ and *F*_RB_. *F*_B_, but not *F*_RB_, was significantly related to the size of the fish (*L*_F_) (Fig 5). It was also found that *W*_G_ was significantly dependent on *L*_F_ (*F*_(1,52)_ = 10.7, r^2^ = 0.1549, *P* <0.01).

**Table 4:** Summary of the regression analyses testing the relationships between batch fecundity (*F*_B_ in million oocytes) and relative batch fecundity (*F*_RB_ in oocytes g^-1^ of somatic-gutted weight) with fork length (*L*_F_, cm), total gonad weight (*W*_G_, g), somatic-gutted weight (*W*_S_, g) and gonadosomatic index (*I*_G_) for albacore tuna in the western Indian Ocean.

	Parameters	r^2^	*P* value	*F*
*F*_B_	*L*_F_	0.1089	<0.05	6.36
	*W*_G_	0.3517	<0.0001	28.2
	*W*_S_	0.0588	0.0774	3.25
	*I*_G_	0.3196	<0.0001	24.4
*F*_RB_	*L*_F_	0.0225	0.2789	1.20
	*W*_G_	0.2667	<0.0001	18.9
	*W*_S_	0.0009	0.8326	0.05
	*I*_G_	0.2978	<0.0001	22.1

## Discussion

### Sex ratio

Sex ratio analysis showed a predominance of males at larger length classes as also observed in the Pacific and Atlantic Oceans [24,29,32,52,53] and for other tuna species such as bigeye tuna (*Thunnus obesus*), yellowfin tuna, Atlantic (*Thunnus thynnus*) and southern bluefin (*Thunnus maccoyii*) tunas [19]. Two main hypotheses have been put forward to explain this: differences in natural mortality [19,54] as a result of higher energetic demand in females when spawning [55], and differential growth rates [56]. Indeed, in South Pacific albacore, length-at-age was found to vary between sexes with males having higher maximum sizes than females [56]. Also similarly to the South Pacific albacore [24], female albacore in the western Indian Ocean tend to dominate the smaller length classes prior to those dominated by males, which is thought to be mostly the effect of the differences in growth rates [57] and could reflect difference in energetic needs for reproduction between sexes [58]. South of 30°S, in South Africa waters, no significant differences in sex ratio were observed at the same length classes where the majority of fish were small and immature, and not yet involved in reproduction.

### Size-at-maturity

The largest fish classified as immature in our study had an *L*_F_ of 94 cm which is the same as estimated for albacore in the South and North Pacific [23,30]. The minimum size-at-maturity found was 83 cm *L*_F_ which is slightly smaller than that reported for North and South Pacific albacore (78–90 cm *L*_F_; [23,24,32,52] and previous estimates for the Indian Ocean albacore (90 cm *L*_F_; [59,60]), but larger than in the Mediterranean Sea (56 cm *L*_F_; [26]). The present study is the first to calculate the *L*_50_ for albacore tuna in the Indian Ocean based on histological analyses, with estimates of the *L*_50_ at 85.3 ± 0.7 cm based on a vitellogenic 3 maturity threshold. Due to the small number of immature fish found in our analysis and the fact that the albacore were caught by different fishing gears, a spatial analysis of size-at-maturity could not be estimated such as for the South Pacific albacore [30]. However, our *L*_50_ is slightly lower than theirs which was estimated at around 87 cm also based on a vitellogenic 3 maturity threshold. To obtain more accurate information on the maturation process, further studies could be designed to account for potential variability in maturity spatially.

Since it was previously suggested that the cortical alveoli stage is a sign of maturation [42], we estimated a new *L*_50_ (84.9 ± 0.8 cm) based on this assumption. However, at present, there is no standard method that allows for the classification of fish as immature or mature [61] and different studies use different oocyte maturity thresholds [24,41,44]. Thus, information on the amount of individual growth that occurs during the time lag between the cortical alveoli and vitellogenic oocyte stage of a species could improve the accuracy of *L*_50_ estimates by defining the most appropriate level of gonad development to be used as maturity threshold.

### Spawning behavior

Histological analyses revealed that the latitudes south of 30°S are inhabited mainly by immature fish while the regions north of 30°S are mostly occupied by mature fish. Spawning individuals were exclusively located between 10°S and 30°S mainly to the east of Madagascar (region C), although evidence of spawning activity was also observed on the western side in the Mozambique Channel (region B). Spawning concentrated from October to January with peak spawning observed in November and December. Our results agree with previous studies which suggested that spawning of albacore in the Indian Ocean occurs between 10°S and 30°S [16,17,59] and in the waters off eastern Madagascar [62,63]. Spawning of albacore was also found to occur at similar latitudes and season in the South Pacific Ocean [24,52,64]. However, spawning in the northeast Pacific extends from March to September from 10°N to 20° N [23]. Albacore peak spawning period in the present study was further supported by high *I*_G_ (>1.5) which corresponded to fish capable of reproducing [65]. The observation of POFs in ovaries of fish from region C was an additional indication of recent spawning activity [66]. The spawning period corresponded to waters with seawater temperatures above 24°C at the latitudes 10–30°S as previously reported for albacore and other tuna species [17,19,67]. The lower *I*_G_ (<1.5) observed in October and January could be related to lower spawning activity at the start and at the end of the spawning season as a result of an asynchrony in spawning at the population level. This can be explained by the gradual increase in the percentage of fish spawning (from October) and falling off (from January) at the end of the spawning season, as observed in the South Pacific albacore [24].

The increase in the proportion of females with regressing ovaries is evidence of the end of spawning season [42] when fish species with indeterminate fecundity, such as albacore, are characterized by an increasing amount of oocytes in atresia [58]. During the spawning season, oocytes are continuously being produced which are recruited into the stock of oocytes in vitellogenesis and this creates an excess of oocytes that are reabsorbed through the process of atresia as the fish reaches the end of the reproductive season [68]. This down-regulation enables fish to adjust the number of oocytes produced relative to their energy reserves [69]. As the fish progresses throughout the spawning season, the incidence of the lower atretic stage (1) decreases while the higher atretic stages (2–4) appear as the vitellogenic oocytes are being reabsorbed gradually during the regressing phase. In the present study, regressing fish with atretic stage 2 were seen mostly during or right after the spawning season while those of atretic stages 3–4 occurred mostly after the spawning season, as from April, showing clearly the evolution of atresia with time. Similar observations were observed in the South Pacific Ocean, where alpha and beta atresia occurred in ovaries of albacore mostly during or right after the spawning season, following which they showed a decreasing trend [30]. Oocyte atresia can then be only observed in their advanced stages (gamma/delta) as yellow bodies, used as a maturity marker in regenerating ovaries through histology in both the Indian Ocean and South Pacific albacore [24,30]. This progression of females from the regressing into the regenerating phase increased gradually in favor of the latter after the spawning season in January, dominating from March to August. In the South Pacific albacore [24], a similar trend in proportion of regenerating females could be detected after the spawning season.

**Fig 6:**
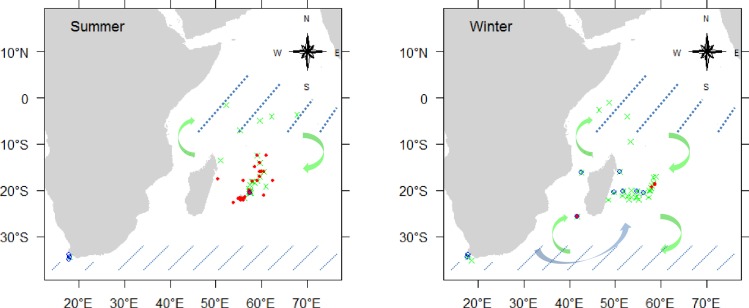
Schematic map showing the distribution and migration of albacore in the western Indian Ocean during austral summer (left) and winter (right). Positions are shown for spawning capable females (red dots), mature inactive (regressing or regenerating phases) females (green crosses) and immature (immature and developing phases) females (blue circles). Diagonal blue dotted lines indicate main location and feeding grounds of immature (thinner lines) and mature (thicker lines) albacore. Arrows indicate movement of mature inactive fish (green arrows) and immature fish (blue arrows).

Fig 6 shows the spatial distribution of immature, spawning capable and mature (inactive) albacore life-history stages in austral summer (October to March) and winter period (April to August) and their likely migration. The distribution is in agreement with previous studies in the Indian [16,17] and South Pacific [24] Oceans. The length-frequency data of albacore also show the presence of smaller-sized fish south of 30°S while larger ones are situated northwards. However, histology reveals that a small proportion of mature individuals were found south of 30°S and some immature fish were caught at the spawning latitudes (10°S to 30°S) especially during winter, from April to July. [70] also revealed the presence of few immature fish at the spawning latitudes outside the spawning period; the authors suggested a recent migration of immature fish during austral winter to feed or to prepare for their first spawning in the following season. In region D, all mature fish were mature inactive (in the regenerating phase), also observed by [24] south of 30°S in the Pacific Ocean. Despite the small number of samples analyzed histologically in this region, the abundance of these mature inactive females appeared lower during the spawning season, which could suggest that mature females have migrated northward of 30°S to the spawning latitudes to reproduce.

North of 10°S, the percentage of females with the different ovarian phases from August to November remains unknown as no fish from this period and area could be sampled. However, we found that most of the fish had ovaries in the regressing phase, even during the spawning months of December and January, suggesting that large-scale spawning does not occur in this region. The presence of mature inactive (regressing and regenerating) females north of 10°S in Seychelles waters and south of 30°S in South African waters, suggests that after the completion of the spawning season, some females migrate out of the spawning latitudes towards the north or the south of the Indian Ocean. This suggested migration is consistent with the decreased catch rates of albacore observed after the spawning season in Mauritius [7] and Reunion Island [4], and the increased catch rates around south of 30°S from April to September [71]. It is likely that the regions located north of 10°S, where nutrient-rich waters occur as a result of upwelling [72] could be used as a main feeding ground by mature albacore to replenish their energy reserves after reproduction. The cold and rich waters south to Madagascar and off South Africa could also be a potential feeding zone for albacore as previously thought for swordfish [73]. This could especially be true for the immature which seem to occur mostly in this area. However, the low proportion of inactive but mature female albacore caught throughout the year at latitudes 10–30°S, in addition to fishery statistics from longliners indicating that albacore are caught in this area throughout the year [8], suggest that part of the females also remain at these latitudes even after the spawning season.

The occurrence of regressing or regenerating females during the spawning period in region C suggests that not all fish spawn during the whole period which is consistent with the asynchrony in spawning activity of the population. This asynchrony could be the result of the difference in spawning period between small and large fish. Previous studies have suggested that larger and older female albacore have a longer spawning period [24]. This difference has also been observed in yellowfin tuna [67], skipjack tuna [74], and swordfish [75].

In this study, a higher proportion of smaller-sized females already had their ovaries in the regressing and regenerating phases compared to larger females (which were mostly in the spawning capable phase) during the same period. These results are consistent with the theory that smaller and younger fish that are first maturing will tend to have a lower spawning activity than larger and older fish [34]. This may in turn be due to the energy equilibrium between somatic growth and reproduction which is dependent on fish size [76]. A shorter spawning period for the small and young fish has also been observed in the Pacific mackerel (*Scomber japonicus*) [77] and jack mackerel (*Trachus symmetricus*) [78]. It was also proposed that the adjustment of small and young mature albacore to the tropical waters could result in a lower spawning fraction [24].

### Spawning frequency and fecundity

The mean interval between two successive spawning events for albacore in the entire western Indian Ocean (3.4) and specifically for region C (2.2) during the spawning period (from November to January) was lower than for the South Pacific albacore during its peak spawning months (1.3 days from October to December) from 10–25°S [24]. The values are also lower than that reported for albacore from the North Pacific Ocean during peak spawning activity (1.7 days) [23] although lower values of 3.0 days have been observed from March to April [53]. The Indian Ocean albacore is characterized by a lower spawning frequency compared to skipjack (1.2 days) [49], yellowfin (1.5 days) [55], bigeye (1.1 days) [79] and southern bluefin tunas (1.6 days) [80]. In albacore, POFs have previously been observed in ovaries with oocytes at maturation and hydration stages [23,24]. Usually, the presence of POFs and oocytes in advanced stages of maturation in an ovary implies that the fish is capable of daily spawning [19]. In the present study, the majority of POFs were observed in ovaries of fish with vitellogenic 3 as most advanced oocytes which is different to that for fish from the North and South Pacific Ocean where POFs were observed in ovaries with migratory nucleus and hydrated oocytes [23,24]. The low spawning frequency estimate for Indian Ocean albacore suggests that their oocyte maturation rate could be lower than those of albacore from the South Pacific. This could in turn be due to lower energy available for reproduction in the region since spawning intervals have been linked to food availability [81]. Further investigations are required to account for the differences in spawning frequencies taking into account possible effects of fishing gear [23].

The mean *F*_RB_ of 53.4 ± 23.2 oocytes per gram of body weight estimated in this study is in the same range as for the North Pacific albacore (50.5 ± 22.8; [23]), southern bluefin tuna (56.5 ± 16.1; [80]), South Pacific albacore (64.4 ± 24.7; [24]) and the Atlantic bluefin (74.7 ± 45.3; [82]). It is also consistent with *F*_RB_ estimated for other tunas such as the western Pacific bigeye (16–150; [83]), yellowfin from the western Indian Ocean (9–180; [67]), the eastern Pacific (68.0 ± 20.7; [43]), the western Pacific Ocean (22.2–77.6; [84]) and Hawaii area (31.9–147.1; [84]), but was lower than the Indian Ocean skipjack tuna (140 ± 64; [74]). In the present study, *F*_B_ for albacore was 0.26 to 2.09 million oocytes, which is higher than the North Pacific albacore (0.17 to 1.66 million eggs; [23]) but is similar to that from the South Pacific albacore although the latter could reach a higher maximum value (0.26 to 2.83 million oocytes; [24]).

It is known that fecundity varies within individuals, being proportional to fish size [34]. It has been suggested that larger and older fish will tend to produce larger numbers of eggs that will hatch into a larger proportion of viable larvae [85]. In our study, *F*_B_ showed a significant relationship with fish size but not with body weight, as is also the case with swordfish [86], and *F*_RB_ is also not significantly related to *L*_F_ as observed in the Atlantic bluefin tuna [82]. This suggests that the increased *F*_B_ in larger albacore is mainly due to their larger ovary as gonad growth is proportionally related to fish size [82]. This is consistent with the result found in the present study which shows that the larger fish developed significantly larger gonads. However, the observed relationship between *F*_B_ and fish size was variable with *F*_B_ of fish of the same size varying to a large extent. This has also been shown in the South Pacific albacore [24] and other tunas [55,79,80] and has been attributed to the period in the spawning season that the fish was caught [24,80]. This is in turn due to the variation in the number of oocytes that are recruited and spawned as the spawning season continues [87]. *F*_B_ has been observed to decrease over time for South Pacific albacore, with higher batches occurring at the beginning than at the end of the spawning season [24]. Fish condition is also a factor that can affect fecundity [34] with better condition linked with higher fecundity [88], earlier maturation and higher energy allocation to egg production [89]. However, reduced oocyte growth has been observed in females with poor feeding conditions [90]. Exploration on the performance of albacore offspring in relation to maternal age and condition could provide additional information that may possibly be integrated in the analysis of the albacore population.

## Conclusions

This study describes numerous aspects of the reproductive biology of albacore in the western Indian Ocean and provides a number of parameters for improving future stock assessments of this species in the region. This will, in turn, enhance the confidence of population estimates of Indian Ocean albacore. Most importantly, stock assessments can be improved by taking into account the bias in sex ratio towards males in the larger size classes, and the importance of large females, and their greater contribution to reproduction, and thus recruitment, through their longer spawning period compared to small individuals. In the long-term, the results could be useful to generate advice and development of management strategies to maintain the MSY of the Indian Ocean albacore stock.

## Supporting Information

S1 Table Number of fish per oocyte developmental stage or ovarian phase by 3-cm interval fork length (*L*_F_) used for maturity analysis of female albacore tuna in the western Indian Ocean based on histology. In the first criterion, immature fish were viewed as containing the most advanced oocyte stage the primary growth (PG) stage, cortical alveolar (CA) and vitellogenic 1 (Vtg1). Mature fish were those containing secondary vitellogenic 2 (Vtg2), vitellogenic 3 (Vtg3), germinal vesicle migration (GVM), germinal vesicle breakdown (GVBD) and hydrated (Hyd) oocytes as most advanced stage, as well as those in regressing (RGP) and regenerating phases (RGNP). In the second criterion, the maturity threshold was set at CA oocyte stage.
(PDF)
