# Supplementary material for: Reproductive Biology of Albacore Tuna (Thunnus alalunga) in the Western Indian Ocean
Source: PLoS One. 2016 Dec 21;11(12):e0168605. doi: 10.1371/journal.pone.0168605 (PMC5176184; doi:10.1371/journal.pone.0168605)
Supplement: S1 Table — In the first criterion, immature fish were viewed as containing the most advanced oocyte stage the primary growth (PG) stage, cortical alveolar (CA) and vitellogenic 1 (Vtg1). Mature fish were those containing secondary vitellogenic 2 (Vtg2), vitellogenic 3 (Vtg3), germinal vesicle migration (GVM), germinal vesicle breakdown (GVBD) and hydrated (Hyd) oocytes as most advanced stage, as well as those in regressing (RGP) and regenerating phases (RGNP). In the second criterion, the maturity threshold was set at CA oocyte stage. (PDF) [file pone.0168605.s001.pdf]

**Ovarian phase**	**IP**	**DP**			**SCP**				**RGP**	**RGNP**	**Total per L_F class**
**Oocyte stage**	**PG**	**CA**	**Vtg1**	**Vtg2**	**Vtg3**	**GVM**	**GVBD**	**Hyd**			
***L_F* classes (cm)** 77-79	3	0	0	0	0	0	0	0	0	0	3
80-82	2	0	0	0	0	0	0	0	0	0	2
83-85	6	0	0	0	0	0	0	0	3	1	10
86-88	4	0	1	0	0	0	0	1	5	11	22
89-91	6	2	0	0	3	1	0	1	26	21	60
92-94	3	0	0	0	7	2	3	1	23	44	83
95-97	0	0	0	0	31	3	7	8	28	81	158
98-100	0	0	0	0	57	2	8	7	19	77	170
101-103	0	0	0	0	46	2	1	4	17	45	115
104-106	0	0	0	0	18	2	0	3	2	8	33
107-109	0	0	0	0	3	0	0	0	3	2	8
110-112	0	0	0	0	0	0	0	0	0	1	1
**Total per oocyte stage / ovarian phase**	24	2	1	0	165	12	19	25	126	291	665
